# Reproducibility of tumor budding assessment in pancreatic cancer based on a multicenter interobserver study

**DOI:** 10.1007/s00428-020-02987-2

**Published:** 2020-12-17

**Authors:** Eva Karamitopoulou, Irene Esposito, Inti Zlobec, Andrea Cacciato Insilla, Martin Wartenberg, David F. Schaeffer, Steve Kalloger, Stefano La Rosa, Christine Sempoux, Irene Ramos Centeno, Philipp Lohneis

**Affiliations:** 1 Pancreatic Cancer Research Group, Institute of Pathology, University of Bern, Bern, Switzerland; 2 Institute of Pathology Heinrich-Heine University & University Hospital, Duesseldorf, Germany; 3 Department of Surgical, Medical and Molecular Pathology and Critical Care Medicine, University of Pisa, Pisa, Italy; 4 Department of Pathology & Laboratory Medicine, University of British Columbia and Division of Anatomic Pathology, Vancouver General Hospital, Vancouver, Canada; 5 Institute of Pathology, University Hospital and University of Lausanne, Lausanne, Switzerland; 6 Faculty of Medicine and University Hospital Cologne, Institute of Pathology, University of Cologne, Cologne, Germany

**Keywords:** Pancreatic cancer, Tumor budding, Interobserver, Prognosis

## Abstract

Tumor budding has been reported to be an independent prognostic factor in pancreatic ductal adenocarcinoma (PDAC). Its use in daily diagnostics would improve the prognostic stratification of patients. We performed a multicenter interobserver study to test various budding assessment methods for their reproducibility. Two serial sections of 50 resected, treatment-naïve PDACs were stained for Hematoxylin and Eosin (H&E) and pancytokeratin. Tumor budding was scored by independent observers at five participating centers in Switzerland, Germany, and Canada. Pathologists assessed tumor budding on a digital platform comparing H&E with pancytokeratin staining in 10 high-power fields (10HPF) and one HPF hotspot (1HPF). Additionally, tumor budding was assessed in one H&E hotspot at × 20 magnification, as suggested by the International Tumor Budding Consensus Conference (ITBCC). Correlation coefficients for bud counts between centers ranged from *r* = 0.58648 to *r* = 0.78641 for H&E and from *r* = 0.69288 to *r* = 0.81764 for pancytokeratin. The highest interobserver agreement across all centers was observed for pancytokeratin 10HPFs (ICC = 0.6). ICC values were 0.49, 0.48, 0.41, and 0.4 for H&E in 1HPF hotspot, H&E in 10HPFs, pancytokeratin in 1HPF, and H&E in one hotspot at ×20, respectively (ITBCC method). This interobserver study reveals a range between moderately poor to moderate agreement levels between pathologists for the different tumor budding assessment methods in PDAC. Acceptable levels of agreement were reached with the pancytokeratin 10HPF method, which can thus be recommended for the assessment of tumor budding in PDAC resection specimens. To improve the levels of interobserver agreement, the implementation of machine learning applications should be considered.

**Supplementary Information:**

The online version contains supplementary material available at 10.1007/s00428-020-02987-2.

## Introduction

Pancreatic adenocarcinoma (PDAC) is a major cause of cancer-associated mortality in Western countries and it is expected to emerge as the second leading cause of cancer-related death by 2030 [1]. Surgical resection with curative intent is currently considered to be the only chance for improving survival [2, 3]. Recent advances in the multimodal management of patients with PDAC have improved the 5-year overall survival rates up to 20–40% following oncologic resection for PDAC [4, 5]. Despite these significant advances in the treatment of PDAC, tumor recurrence following radical resection remains high thus limiting long-term survival [6]. Furthermore, PDAC is a highly heterogeneous disease and even patients with the same TNM stage have different outcomes [7]. Thus, the identification of biomarkers that would enable a more accurate prediction of the tumor biology of PDAC is necessary, in order to optimize modern individualized patient management.

Tumor buds are defined as single or small groups of up to four carcinoma cells growing detached from the main tumor [8] and have been shown to display features of epithelial-to-mesenchymal transition as well as stem cell features [9–12]. Increased numbers of tumor buds have been correlated with adverse clinicopathological features, such as diminished progression-free and overall survival in many gastrointestinal cancers [13]. Since tumor budding has been recognized as a biomarker with prognostic significance in pancreatic ductal adenocarcinoma (PDAC) [14–17], including information on a parameter such as tumor budding into the histopathology reports would supply an additional useful tool in adjusting personalized patient risk stratification. Thus, information regarding tumor budding could provide a prognostic indicator to help in the identification of high-risk patients who would profit from a more intensified tumor surveillance following oncologic resection with curative intent. However, although tumor budding is considered a promising and/or additional prognostic factor for other tumor entities, such as colorectal cancer [18], it is still not included in any classification or protocol for PDAC and it is not recommended by any society, including the College of American Pathologists.

One of the reasons that might explain the reluctance to report tumor budding in PDAC is the lack of a unified, standardized, and reproducible scoring method. To add confusion, several methods have been used so far for the evaluation of tumor budding, encompassing semiquantitative or quantitative methods with different cut-offs [19–21]. Moreover, some studies assess tumor budding on hematoxylin and eosin (H&E)–stained slides while others use immunohistochemical staining for pancytokeratin, which on one hand greatly increases the number of identifiable tumor buds, but on the other hand, also increases the turnaround times and expenses of specimen evaluation and reporting [22]. More recently, the International Tumor Budding Consensus Conference (ITBCC) 2016 for colorectal cancer proposed the assessment of one hotspot at × 20 magnification on H&E-stained slides as a standardized and easily applicable method to report tumor budding in colorectal cancer [23], which has been validated and reproduced also in PDAC [17, 24]. Another important aspect that hampers the clinical use of tumor budding in PDAC is the issue of interobserver variability. So far, a number of studies have addressed the interobserver variability of tumor budding assessment in colorectal cancer and reported low to acceptable levels of agreement [22, 25, 26], while currently, studies examining the multi-institutional interobserver variability for the various reported tumor budding assessment methods do not exist for PDAC.

The challenge thus lies in the identification of the most reproducible method to assess tumor budding, to help include this information into daily diagnostic practice. We performed a multicenter interobserver study between pathologists at five participating, high-volume diagnostic centers in Switzerland, Germany, and Canada with the objective to test various tumor budding assessment methods in PDAC for their reproducibility. In this, we compared five different methods of evaluation, including the assessment of 10 high-power fields (10HPF) and of one HPF hotspot (1HPF) both using H&E and pancytokeratin staining, as well as the evaluation of one H&E hotspot at × 20 magnification as suggested by ITBCC [23].

## Material and methods

### Patients and tissue blocks

Out of routinely assessed PDAC resection specimens from treatment-naïve patients operated at the Insel University Hospital, Bern, Switzerland, and at University Clinic Cologne, 50 PDACs TNM stage I–III, were randomly selected for the present study. All tumor slides of each case (minimum of one tumor block per 1 centimeter of tumor diameter), were reviewed by an experienced pathologist (either PL or EK). One representative tumor block from the center of the tumor containing pancreatic tissue mostly occupied by carcinoma was selected for further processing. Two 4-μm-thick serial sections were cut: one was stained with H&E while the other underwent immunohistochemistry for the pancytokeratin marker AE1/AE3 (Dako, mouse monoclonal, 1:200) using a Leica Bond III instrument. The evaluation was performed after anonymization following all the ethical guidelines required by all institutions with which all the authors are affiliated. The clinicopathological characteristics of the patients are summarized in Suppl. Table 1. The study design is depicted in Suppl. Figure S1. The Ethics Commission of the Canton of Bern has approved the use of patients’ tissue for the implementation of this project (2019-02212). Informed consent is available for all patients.

### Evaluation of tumor budding

Tumor budding was defined as single cells or clusters of up to four tumor cells present at any tumor area. For pancytokeratin-stained slides, tumor buds needed to show clear cytoplasmic cytokeratin reactivity and a nucleus. Cytoplasmic pseudofragments or areas of necrosis were excluded.

### Digital pathology and selection of areas for counting

All slides (*n* = 2 × 50 = 100) were scanned and digitalized in a 3DHistech P250 scanner and a separate case center account for each participating pathologist was created. The slides were evaluated by virtual microscopy using the Case Viewer software (Case Viewer 3DHISTECH_Ltd Version 2.2.0.85100). Observers were blinded to clinicopathological data and patient information and scored tumor budding in H&E and pancytokeratin-stained slides independently.

**Fig. 1:**
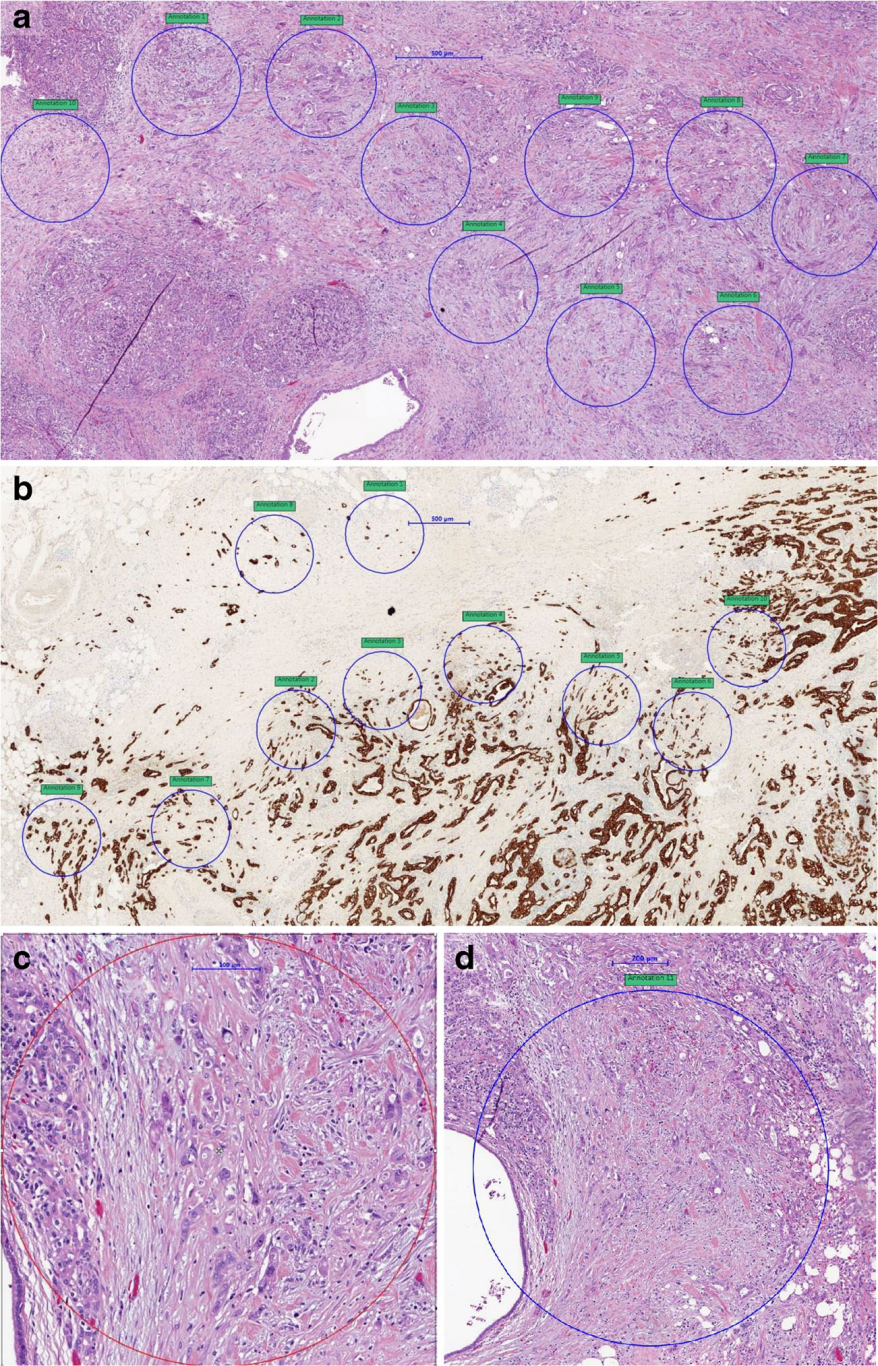
Tumor budding assessment on digital slides: HPFs of standardized size (× 40, field diameter 600 μm, area 282 μm^2^) and at × 20 (field diameter 1100 μm, area 950 μm^2^) were simulated using a fixed size annotation which was placed by each observer independently in areas of highest tumor budding density. The average number of tumor buds counted on H&E- and pancytokeratin-stained slides was calculated and compared for agreement across centers. Overview of 10 HPFs on H&E (a)-and pancytokeratin (b)-stained slides. Higher magnification of 1HPF (× 40 magnification; c) and at × 20 magnification (d) on an H&E-stained slide

Areas of tumor budding were first identified using low power magnification. Standardized counting areas representing one field at × 40 magnification representing 1 high-power field (1HPF; field diameter 600 μm, area 282 μm^2^) and one field at × 20 magnification (field diameter 1100 μm, area 950 μm^2^) were simulated using fixed-size annotations and placed by each observer individually in areas of highest budding density (Fig. 1). Tumor buds were then counted in a total of 10 fields at × 40 magnification (10 HPFs method) and in one densest hotspot at × 40 magnification (1HPF method), both in H&E and pancytokeratin. Then, tumor budding was counted in one hotspot at × 20 magnification in H&E staining (area 950 μm^2^). The number of tumor buds counted in that area was divided by 1.21 to obtain the number of buds in an area of 785 μm^2^ according to the ITBCC method [23]. All counts were recorded in an excel file. Each participating center provided one single excel file with the budding counts. The values were compared for agreement across the five participating centers. Pathologists of institute 1, institute 3, and institute 5 had previous experience, while pathologists of institute 2 and institute 4 had no previous experience in the evaluation of tumor budding in pancreatic cancer.

### Statistics

Descriptive statistics were carried out on tumor budding scores. In a first step, Pearson’s correlation coefficient (*r*) was used to determine the strength of the linear relationship between observers’ values. Then to determine the interobserver variability, the intraclass correlation coefficient (ICC) was used. The ICC may be interpreted similarly to the Kappa with values closer to 1.0 indicating stronger agreement.

## Results

### Comparison between pancytokeratin and H&E counts across the participating centers

Tumor budding was scored by independent observers at five participating centers in Switzerland, Germany, and Canada, on H&E-stained slides and matched pancytokeratin (AE1/AE3)-stained slides of 50 PDAC cases. Representative images of the PDAC slides with the annotations (H&E and pancytokeratin) are depicted in Fig. 1.

Average tumor budding counts across centers ranged from 7 to 11.6 buds using the 10HPF method on pancytokeratin-stained slides (median: 8.7). These values were on average significantly higher than with the 10HPF method on H&E slides, which ranged from 2.4 to 7 buds (median: 4.6; Table 1).

**Table 1:** Descriptive statistics on tumor budding by institute and evaluation method

Scoring method	Institute	Mean	Median	Min	Max
H&E 10 HPFs	1	5.0	3.6	0	22.6
	2	7.0	6.6	1.5	17.0
	3	5.5	4.6	0.4	25.
	4	2.4	1.3	0	14.7
	5	6.0	4.5	0.7	20.7
	All	5.1	4.6	0.8	17.9
Pancytokeratin 10 HPFs	1	8.5	6.9	0	54.4
	2	11.3	10.4	3.2	26.2
	3	9.8	8.9	0.4	38.8
	4	7.0	5.5	0.3	28.7
	5	11.6	8.1	2.3	58
	All	9.7	8.7	2.9	34.7
H&E 1 HPF	1	9.9	8.0	0	43.0
	2	11.5	11.	2.0	30.0
	3	9.7	8.0	1.0	44.0
	4	6.8	4.0	0	42.0
	5	12.5	10.0	4.0	44.0
	All	10.1	9.1	1.4	36.6
Pancytokeratin 1 HPF	1	14.9	12.0	0	73.0
	2	20.9	21.0	8.0	42.0
	3	17.3	15.0	1.0	58.0
	4	15.4	13.0	1.0	62.0
	5	28.7	22.0	5.0	152.0
	All	19.4	16.9	7.0	66.0
H&E 1 hotspot 20xITBCC	1	13.1	11.0	0	73.0
	2	16.3	14.5	4.0	35.0
	3	14.7	11.0	1.0	57.0
	4	11.6	9.0	0	35
	5	18.9	14.0	3.0	62
	All	14.5	13.3	4.6	42.4

A similar trend was observed using the 1HPF hotspot method. The single densest 1HPF containing tumor buds on a pancytokeratin stain ranged from 14.9 to 28.7 (median: 16.9) in comparison to a range from 6.8 to 12.5 buds on H&E (median: 9.1). Again, on the pancytokeratin stain, significantly more tumor buds were identified in comparison to H&E. The average number of tumor buds across centers ranged from 11.6 to 18.9 when scoring tumor buds in 1 hotspot of an H&E stained slide at × 20 (median: 13.3; Table 1).

### Correlation of tumor budding counts between centers

A correlation matrix was performed in order to visualize the relationship between tumor budding scores across all centers (Suppl. Figure S2). Each center was compared to the four others.

For H&E-stained slides, assessed using the 10HPF method, correlation coefficients ranged from *r* ≤ 0.39 to *r* ≤ 0.84, with an average of *r* = 0.76. The 1HPF method for H&E slides performed similarly; the correlations had values of 0.48 to 0.85 and an overall *r* of 0.71. For pancytokeratin assessment of tumor budding using the 10HPF approach, medium to strong correlations were found with values of 0.51 ≤ *r* ≤ 0.84 and an average overall *r* = 0.78. 1HPF in pancytokeratin staining performed worse with a range of values from 0.11 to 0.81 and an overall correlation coefficient of 0.50. The correlations of tumor budding counts observed between centers for the ITBCC scoring method ranged from 0.16 to 0.87 with an average *r* = 0.55. Correlation coefficients for the tumor budding categories (BD1, BD2, and BD3) according to the ITBCC method [23, 24] ranged between 0.1 and 0.78 with an average *r* = 0.44. The best correlation, especially for the ITBCC method (both for buds counts and budding categories), was observed between institutes 1 and 3 (both had previous experience on the assessment of tumor budding by this and other methods; Suppl. Tables 2 and 3).

### Interobserver agreement of tumor budding counts

**Fig. 2:**
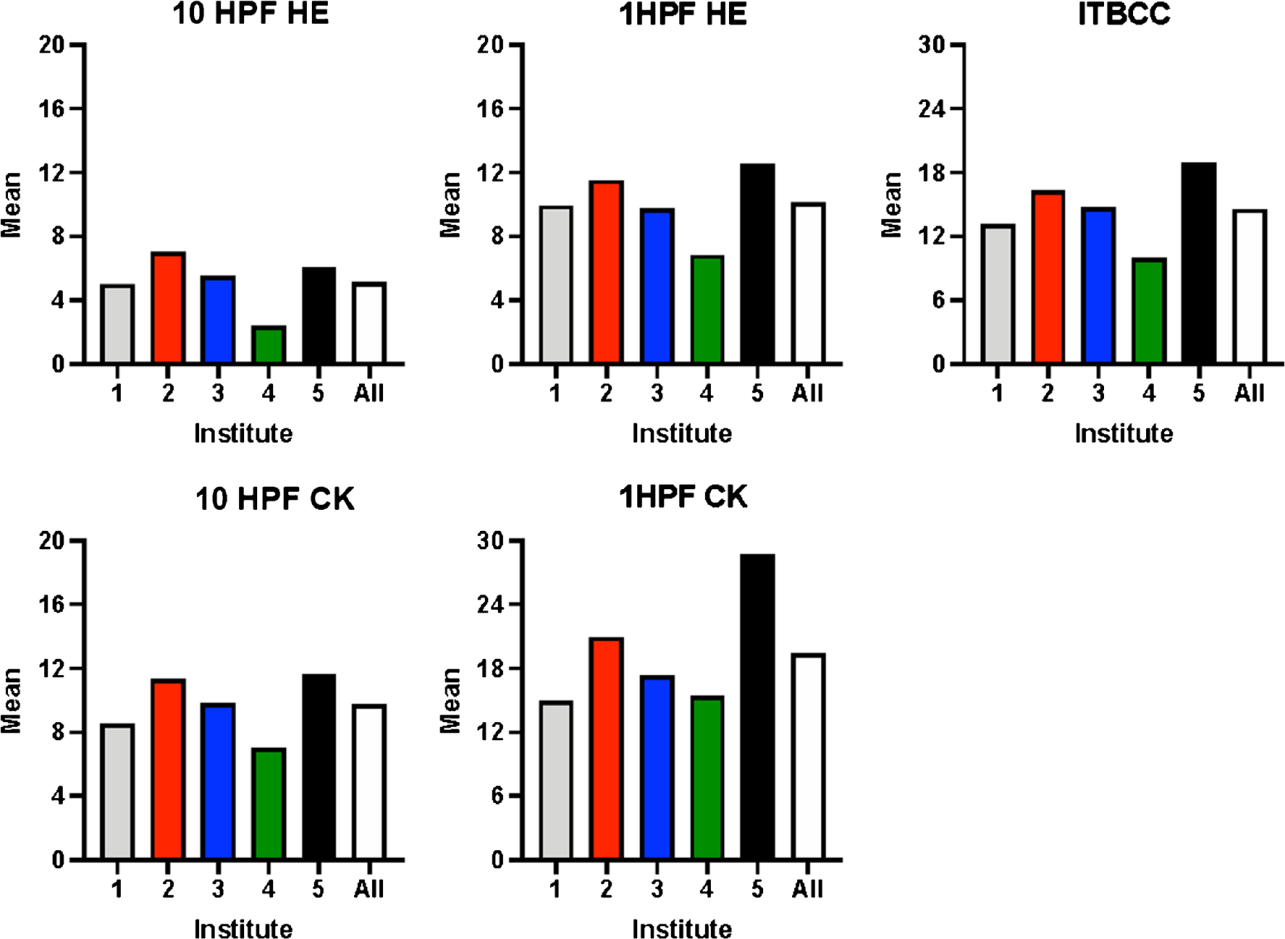
Graphic representation of the mean counts of identified tumor buds across all five institutes for the five evaluation methods

Correlation coefficients as a measure of linear relationship do not represent interobserver agreement. Therefore, to assess the agreement across all centers, the ICC values were calculated. In descending order, ICC values were 0.6, 0.49, 0.48, 0.41, and 0.4 for pancytokeratin in 10HPF method, H&E in 1HPF method, H&E in 10HPF method, and pancytokeratin in 1HPF method and ITBCC method (one hotspot H&E at × 20) respectively (Table 2). A graphic representation of the mean numbers of tumor buds by each evaluation method across all five institutes is shown in Fig. 2.

**Table 2:** Intraobserver agreement using intraclass correlation coefficient (ICC) for tumor budding scores using different methods by institute

Method	ICC
H&E 10 HPFs	0.48
Pancytokeratin 10 HPFs	0.6
H&E 1 HPF	0.49
Pancytokeratin 1 HPF	0.41
ITBCC	0.40

## Discussion

Several factors have been identified to be associated with diminished disease-free survival of the patients after resection of pancreatic cancer including high tumor stage, lymph node metastases [27], tumor involvement of the resection margins [28], and last but not least tumor budding, which has been found to add independent prognostic information [14–17]. All these factors are regularly included in the histopathology reports except tumor budding, which is still missing in most reports of resection specimens of treatment-naïve PDACs. Including information on tumor budding would greatly improve the prognostic stratification of PDAC patients, taking into consideration the heterogeneity of PDAC tumors and the lack of parameters other than TNM that are able to provide such strong and independent prognostic information upon histomorphologic evaluation [14–17]. There can be several reasons for the reluctance to report tumor budding, one major issue being related to the current lack of consensus concerning the optimal evaluation method of tumor budding in PDAC that underlines the need for a standardized and reproducible scoring system.

We undertook an interobserver study in which we compared different methods for the evaluation of tumor budding [21, 23, 29] for their reliability, reproducibility, and applicability. These methods and especially the 10HPFs (either by pancytokeratin or by H&E) and the ITBCC method were chosen because they have already been used to prove the prognostic utility of tumor budding in large series of PDACs [14–17, 24]. By performing this study, we were confronted with all the issues related to such an evaluation, the most important of which will be briefly addressed in the following paragraphs.

One important issue is the selection of the most topographically suitable tumor area to evaluate the tumor buds. Concerning PDAC, many factors support the fact that the evaluation can actually take place across the whole tumor area, independent of the topographic location within the tumor (intratumorally or at the invasive front). Indeed, in PDAC resection specimens, the distinction between intratumoral (i.e., within the main tumor bulk) and peritumoral budding (i.e., at the invasive front) is not always clear, and the borders between them are often blurred. Care has to be taken, however, to distinguish tumor budding from poor differentiation, as tumor budding differs from the tumor grade. Indeed, for the evaluation of tumor grade, several morphologic factors, such as the proportion of tumor gland forming component, mucin production by the tumor cells, the number of mitoses, and nuclear polymorphism, have to be considered [30]. Tumor budding on the other hand is defined as the presence of single cells or clusters of up to four neoplastic cells in a tumor area and although it is more pronounced in poorly differentiated tumors and has been found to correlate with tumor grade [17], it can also be found in better-differentiated carcinomas. Moreover, high-grade tumor budding was proved to be an independent adverse prognostic factor in PDAC, also when tumor grade was considered in the multivariate analysis [15].

Another factor that could primarily affect the reproducibility of tumor budding scoring is the different levels of experience among pathologists [25, 26]. Therefore, care was taken so that pathologists from centers with both previous and no previous experience in the evaluation of tumor budding should participate in this study. Indeed, interobserver agreement was higher among pathologists with previous experience for almost all methods, with only the pancytokeratin 10HPF method showing an acceptable overall interobserver agreement, independently of the experience level of the pathologist who performed the scoring. Especially concerning the ITBCC method, which has been found to represent a very good and cost-effective tool for the assessment of tumor budding in pancreatic cancer [24], a high interobserver agreement was observed only among pathologists with previous experience on assessing tumor budding. This on another note signifies that practice can improve the rates of interobserver agreement for all methods and for the ITBCC method in particular.

A further frequently addressed issue when it comes to the most suitable scoring method is the question about the necessity to perform pancytokeratin staining for the evaluation of the tumor buds. It is true that in almost all studies, including the present one, pancytokeratin staining has been proved superior to H&E concerning the identification of tumor buds, allowing for the recognition of a greater bud number when compared with H&E [22]. Pancytokeratin staining helps, for example, distinguishing tumor buds from activated fibroblasts or small clusters of neuroendocrine cells that may be encountered among the tumor infiltrates. However, the application of an immunohistochemical staining slightly increases the expenses and the time needed for the evaluation and reporting of the specimen. For this reason, we assessed tumor buds by evaluating both pancytokeratin and H&E stained slides and by applying different assessment methods. Indeed, the interobserver agreement after the evaluation of 10 HPFs in pancytokeratin-stained slides achieved the best ICC value (ICC = 0.6) among all participating pathologists. When applying the 1HPF method in pancytokeratin-stained slides, the distribution of scores between centers was considerably more dispersed and the interobserver agreement dropped to 0.41, achieving similar ICC values to that of H&E scoring methods (0.40 to 0.49). This suggests that pancytokeratin staining performs better than H&E only after evaluation of a considerably greater number of high-power fields. Moreover, our results show that the ITBCC method still holds value, but only among pathologists with previous experience in tumor budding evaluation. Therefore, and after taking into consideration important factors such as reproducibility and reliability, the pancytokeratin 10HPF method seems to be the method of choice for the assessment of tumor budding in pancreatic cancer resection specimens from non-neoadjuvantly treated patients. For the assessment of tumor budding in specimens with only limited amount of evaluable tumor material, such as small invasive carcinoma foci in a predominantly non-invasive cystic pancreatic neoplasm, encompassing pT1 carcinomas, the evaluation of 1 HPF in H&E-stained slides, which achieved the second best ICC value in our study (0.49), would be more suitable. Assessing tumor budding in PDAC resection specimens from neoadjuvantly treated patients cannot be recommended at this point and further studies are required for its inclusion, in comparison with stage, regression grading, and outcome.

The present interobserver study on tumor budding evaluation in pancreatic cancer reached only moderate levels of agreement among participating institutes. This is in keeping with other previous interobserver studies on tumor budding assessment in colorectal cancer [25, 26, 31] and can be attributed to various reasons, including the stain used (H&E versus pancytokeratin) and the experience and/or expertise of the pathologists, as discussed in the previous paragraphs. One further parameter is that this and other studies include the use of digital images for the evaluation of tumor budding, in contrast to the microscopic assessment still used for routine diagnostics by most pathologists. Although digital pathology is becoming increasingly part of our lives, the experience levels of pathologists in evaluating digital slides are variable, contributing to the suboptimal interobserver agreement. Recently, an effort is being made to overcome these difficulties by optimizing computer-aided detection systems, which by employing deep learning algorithms would be capable of recognizing complex structures such as tumor buds [31].

Finally, after conducting a multi-institutional, interobserver study using five different scoring methods of tumor budding and taking into account all the above-discussed issues, we can deduce that only the pancytokeratin 10 HPF scoring method achieved acceptable levels of interobserver agreement and thus can be recommended for the assessment of tumor budding in PDAC resection specimens from treatment-naïve patients. To improve the levels of interobserver agreement, the implementation of machine learning applications should be considered.

## Supplementary information

Supplementary Fig. S1 Study Design (PNG 663 kb)

High resolution image (TIFF 446 kb)

Supplementary Fig. S2 Comparison of tumor budding scores across all five centers using H&E and pancytokeratin stains in a correlation matrix. It depicts the correlation on 10 HPFs and 1 densest HPF on H&E and pancytokeratin stained slides, as well as the correlation on one 20x field on H&E (PNG 2004 kb)

High resolution image (TIF 425 kb)

ESM 1 (DOCX 18 kb)
