# Supplementary figures and images for: Reproducibility of tumor budding assessment in pancreatic cancer based on a multicenter interobserver study
Source: Virchows Arch. 2020 Dec 17;478(4):719–26. doi: 10.1007/s00428-020-02987-2 (PMC7990816; doi:10.1007/s00428-020-02987-2)

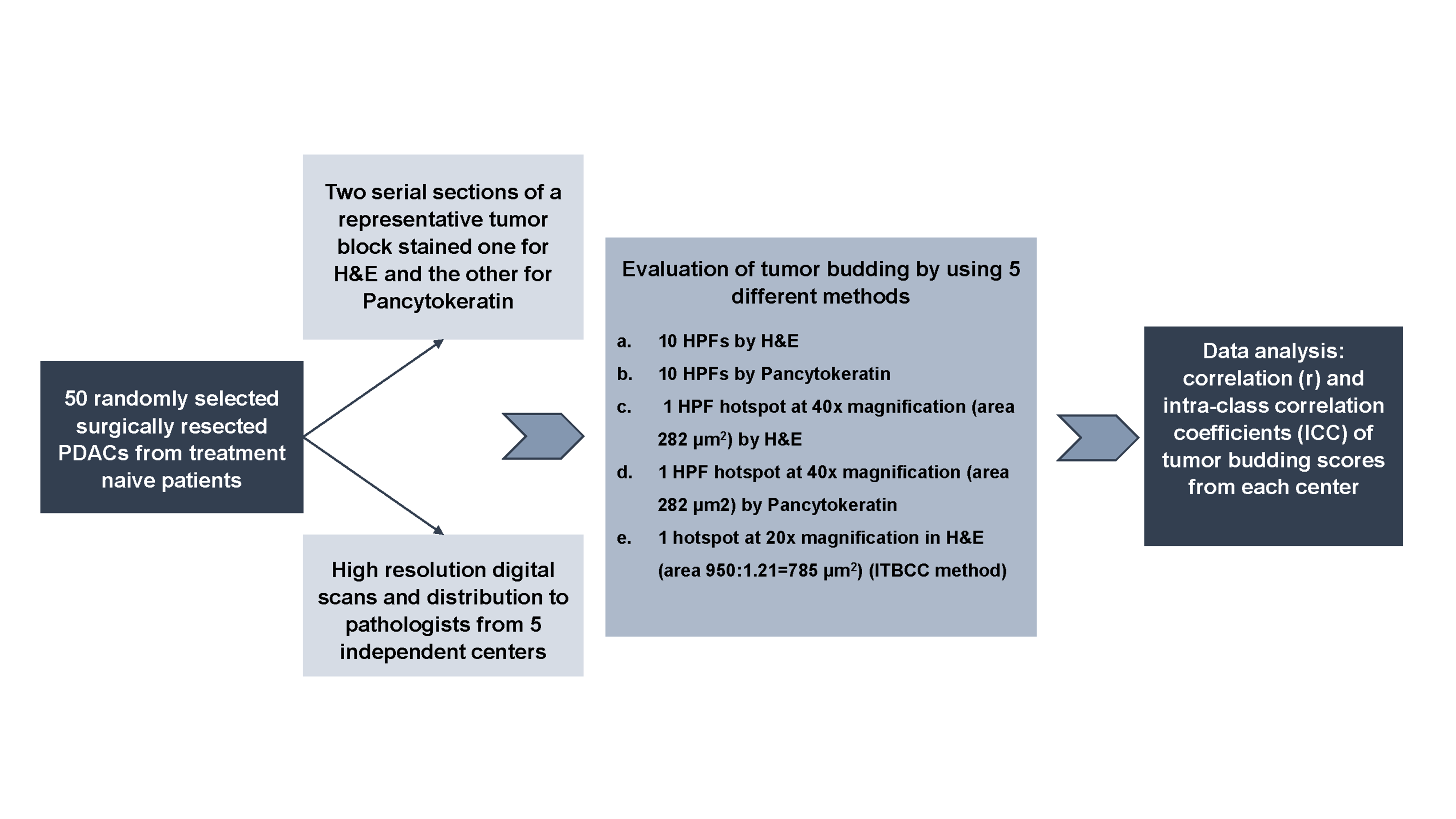

Supplement: Supplementary file 1 — Study Design (PNG 663 kb) [file 428_2020_2987_Fig3_ESM.png]

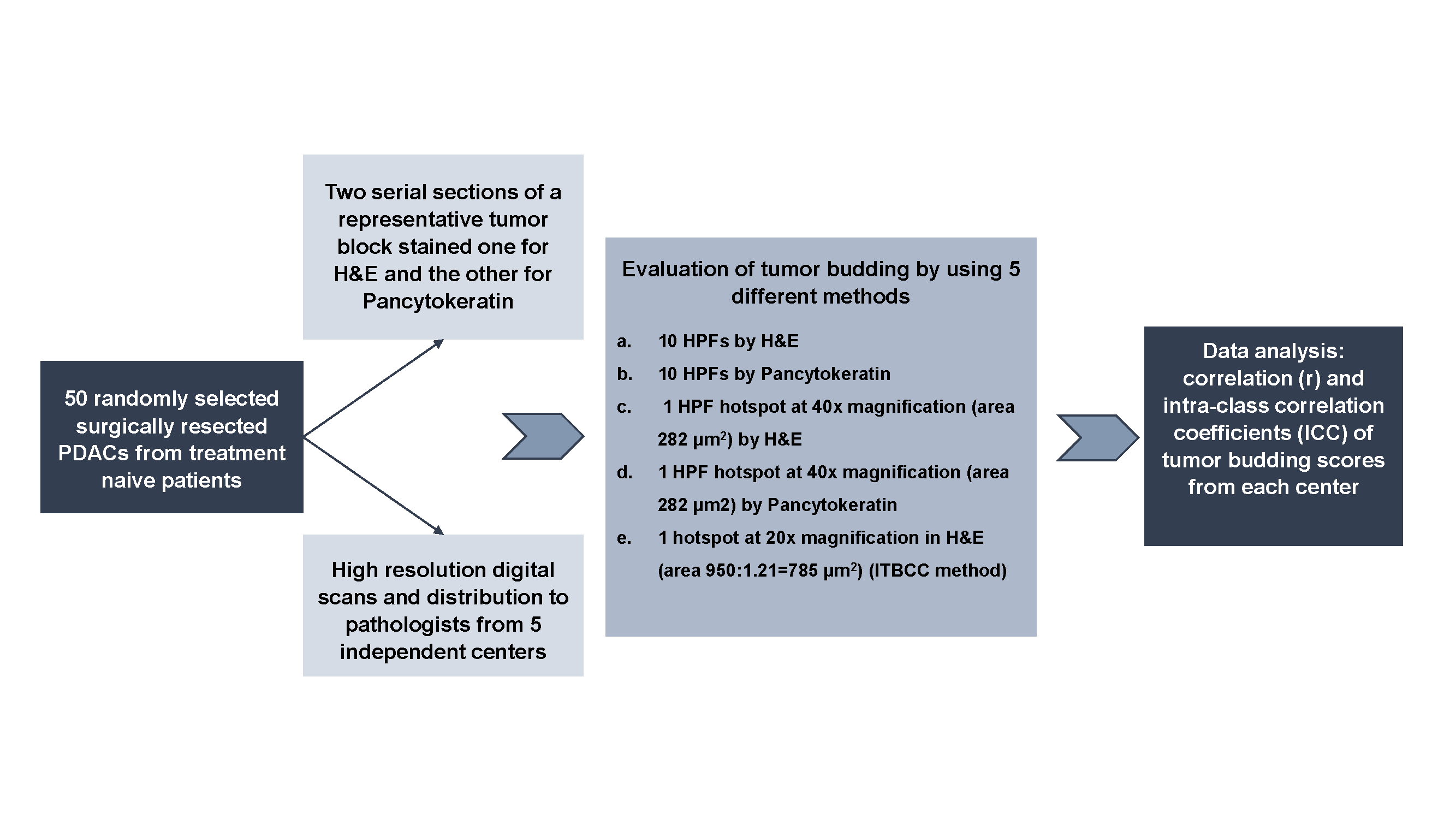

Supplement: Supplementary file 2 — High resolution image (TIFF 446 kb) [file 428_2020_2987_MOESM1_ESM.tiff]

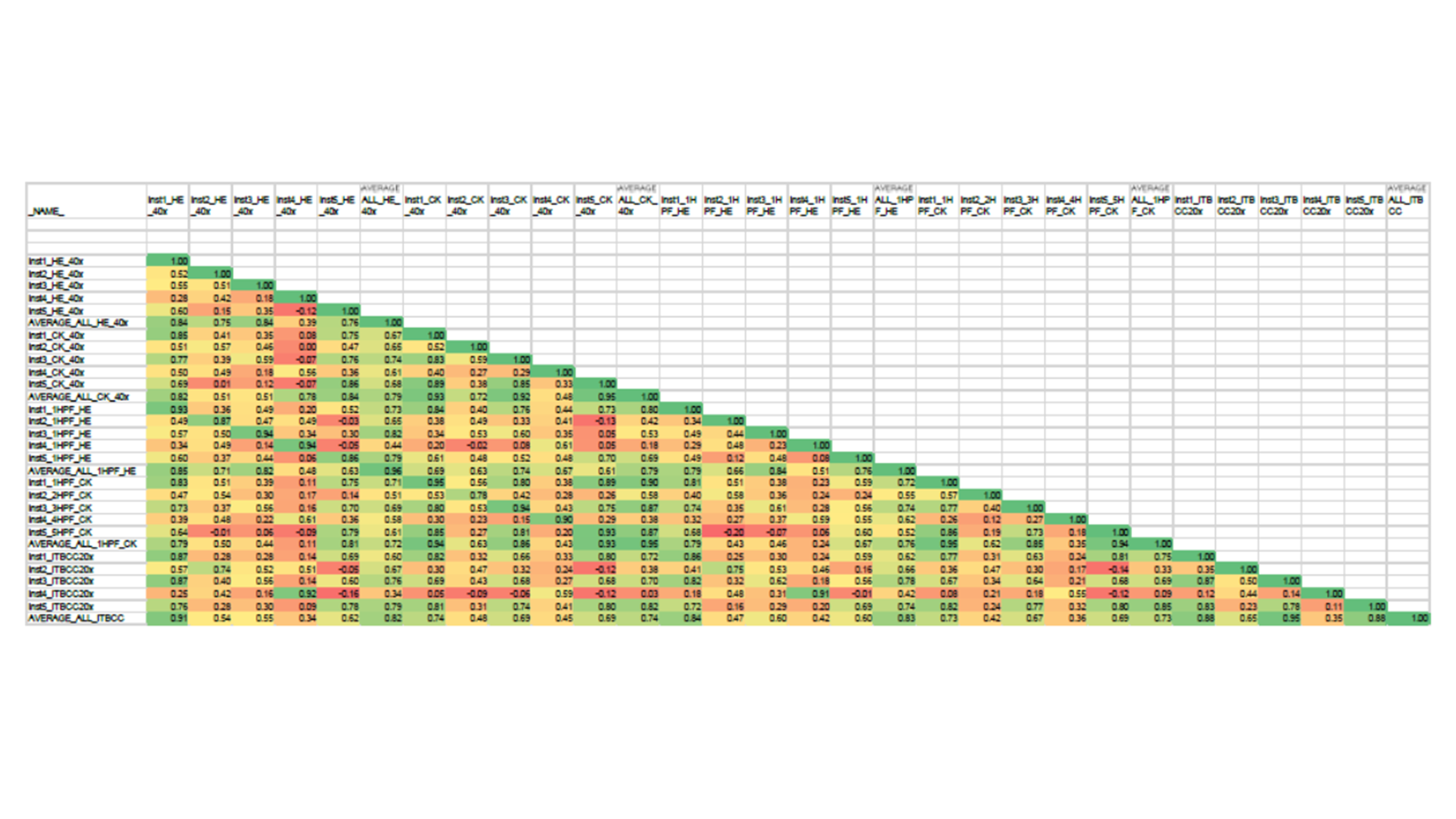

Supplement: Supplementary file 3 — Comparison of tumor budding scores across all five centers using H&E and pancytokeratin stains in a correlation matrix. It depicts the correlation on 10 HPFs and 1 densest HPF on H&E and pancytokeratin stained slides, as well as the correlation on one 20x field on H&E (PNG 2004 kb) [file 428_2020_2987_Fig4_ESM.png]

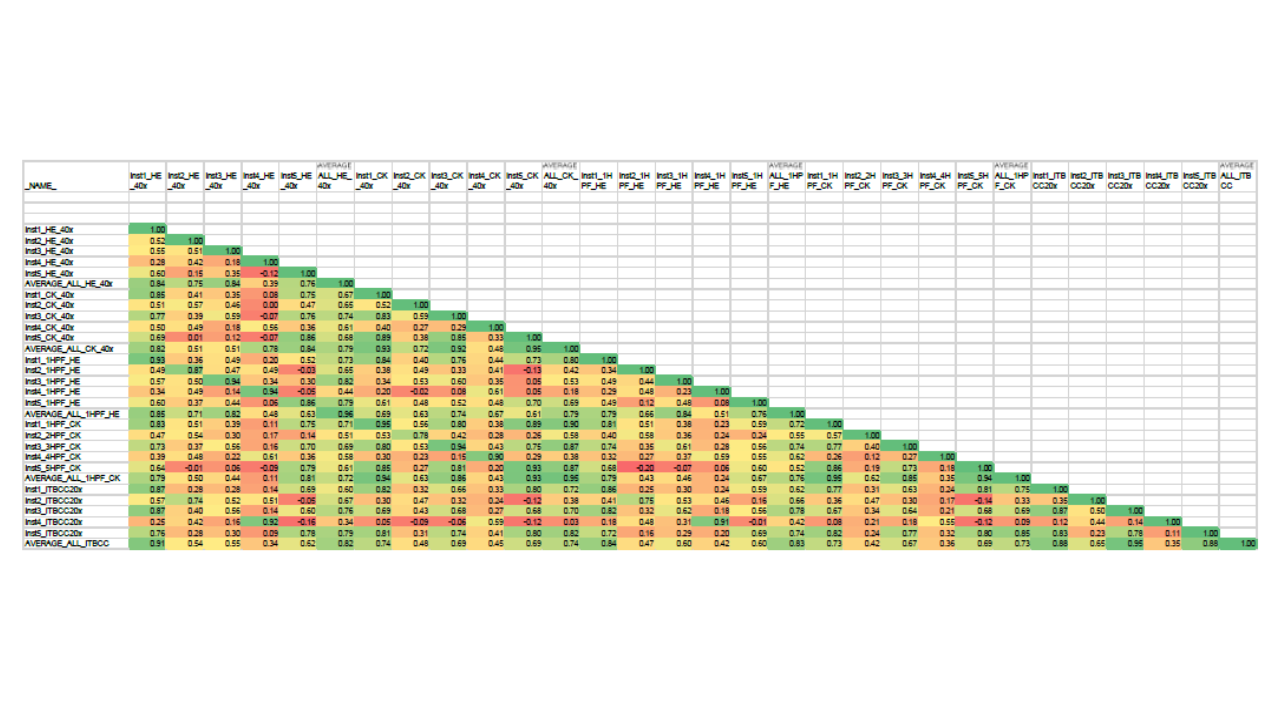

Supplement: Supplementary file 4 — High resolution image (TIF 425 kb) [file 428_2020_2987_MOESM2_ESM.tif]
