# Supplementary material for: Reproducibility of tumor budding assessment in pancreatic cancer based on a multicenter interobserver study
Source: Virchows Arch. 2020 Dec 17;478(4):719–26. doi: 10.1007/s00428-020-02987-2 (PMC7990816; doi:10.1007/s00428-020-02987-2)
Supplement: Supplementary file 5 — (DOCX 18 kb) [file 428_2020_2987_MOESM3_ESM.docx]

**Supp. Table 1:** Clinical Characteristics

| Characteristic | | Number of Patients (%) |
| --- | --- | --- |
| Age | Median (range) | 64.7 (35-84) |
| Sex | M | 30 (60%) |
|  | F | 20 (40%) |
| Size (cm) | Median (Range) | 3.5 (1.2-6.5) |
| Grade | G1 | 3 (6%) |
|  | G2 | 31 (62%) |
|  | G3 | 16 (32%) |
| T-stage | T1 | 5 (10%) |
|  | T2 | 28 (56%) |
|  | T3 | 17 (34%) |
|  | T4 | 0 (0%) |
| N-stage | N0 | 10 (20%) |
|  | N1 | 22 (44%) |
|  | N2 | 18 (36%) |
| M-stage | M0 | 50 (100%) |
|  | M1 | 0 (0%) |
| UICC | IA | 2 (4%) |
|  | IB | 6 (12%) |
|  | IIA | 5 (10%) |
|  | IIB | 22 (44%) |
|  | III | 15 (30%) |
|  | IV | 0 (0%) |
| R-stage | R0 | 28 (56%) |
|  | R1 | 22 (44%) |
| L | L0 | 13 (26%) |
|  | L1 | 37 (74%) |
| V | V0 | 25 (50%) |
|  | V1 | 25 (50%) |
| Pn | Pn0 | 5 (10%) |
|  | Pn1 | 45 (90%) |

**Suppl. Table 2:** Correlations (r) of tumor budding counts between institutes by using the ITBCC method

|  | **Institute** |  |  |  |
| --- | --- | --- | --- | --- |
| **Institute** | **1** | **2** | **3** | **4** |
| 2 | 0.34 |  |  |  |
| 3 | 0.87 | 0.51 |  |  |
| 4 | 0.28 | 0.53 | 0.45 |  |
| 5 | 0.5 | 0.16 | 0.43 | 0.24 |
|  |  |  |  |  |

**Suppl. Table 3:** Summary of (weighted) kappa statistics (k) with 95%CI for the tumor budding categories (BD1, BD2 and BD3) according to the ITBCC method

|  | **Institute** |  |  |  |
| --- | --- | --- | --- | --- |
| **Institute** | **1** | **2** | **3** | **4** |
| 2 | 0.23 |  |  |  |
| 3 | 0.78 | 0.23 |  |  |
| 4 | 0.32 | 0.1 | 0.24 |  |
| 5 | 0.24 | 0.11 | 0.18 | 0.1 |
|  |  |  |  |  |
